# Metformin Reduces Histone H3K4me3 at the Promoter Regions of Positive Cell Cycle Regulatory Genes in Lung Cancer Cells

**DOI:** 10.3390/cancers13040739

**Published:** 2021-02-10

**Authors:** Dongho Kim, Yujin Kim, Bo Bin Lee, Eun Yoon Cho, Joungho Han, Young Mog Shim, Duk-Hwan Kim

**Affiliations:** 1 Department of Molecular Cell Biology, Samsung Biomedical Research Institute, Sungkyunkwan University School of Medicine, Suwon 16419, Korea; haer96@skku.edu (D.K.); yujin0328@hanmail.net (Y.K.); whitebini@hanmail.net (B.B.L.); 2 Samsung Medical Center, Department of Pathology, Sungkyunkwan University School of Medicine, Seoul 06351, Korea; eunyoon.cho@samsung.com (E.Y.C.); joungho.han@samsung.com (J.H.); 3 Samsung Medical Center, Department of Thoracic and Cardiovascular Surgery, Sungkyunkwan University School of Medicine, Seoul 06351, Korea; youngmog.shim@samsung.com

**Keywords:** lung cancer, metformin, H3K4 methylation, MLL2, cell cycle

## Abstract

**Simple Summary:**

To understand the effect of metformin on epigenetic regulation, we analyzed histone H3 methylation, DNA methylation, and chromatin accessibility in lung cancer cells. Metformin showed little effect on DNA methylation or chromatin accessibility but significantly reduced H3K4me3 levels at the promoters of positive cell cycle regulatory genes. Metformin downregulated H3K4 methyltransferase MLL2 expression and knockdown of MLL2 resulted in suppression of H3K4me3 expression and lung cancer cell proliferation. We further evaluated the clinicopathological significance of MLL2 in tumor and matched normal tissues from 42 non-small cell lung cancer patients. MLL2 overexpression was significantly associated with poor recurrence-free survival in lung adenocarcinoma. Our study facilitates the understanding of the effect of metformin on the regulation of histone H3K4me3 at promoter regions of cell cycle regulatory genes in lung cancer cells, and MLL2 may be a potential therapeutic target for lung cancer therapy.

**Abstract:**

This study aimed at understanding the effect of metformin on histone H3 methylation, DNA methylation, and chromatin accessibility in lung cancer cells. Metformin significantly reduced H3K4me3 level at the promoters of positive cell cycle regulatory genes such as CCNB2, CDK1, CDK6, and E2F8. Eighty-eight genes involved in cell cycle showed reduced H3K4me3 levels in response to metformin, and 27% of them showed mRNA downregulation. Metformin suppressed the expression of H3K4 methyltransferases MLL1, MLL2, and WDR82. The siRNA-mediated knockdown of MLL2 significantly downregulated global H3K4me3 level and inhibited lung cancer cell proliferation. MLL2 overexpression was found in 14 (33%) of 42 NSCLC patients, and a Cox proportional hazards analysis showed that recurrence-free survival of lung adenocarcinoma patients with MLL2 overexpression was approximately 1.32 (95% CI = 1.08–4.72; *p* = 0.02) times poorer than in those without it. Metformin showed little effect on DNA methylation and chromatin accessibility at the promoter regions of cell cycle regulatory genes. The present study suggests that metformin reduces H3K4me3 levels at the promoters of positive cell cycle regulatory genes through MLL2 downregulation in lung cancer cells. Additionally, MLL2 may be a potential therapeutic target for reducing the recurrence of lung adenocarcinoma.

## 1. Introduction

Lung cancer is the leading cause of cancer-related death worldwide. Despite the development of new therapeutic agents, the overall five-year survival rate of non-small cell lung cancer (NSCLC) is still less than 20% [1]. Epigenetic and genetic changes play an important role in the development and progression of lung cancer. Epigenetic modifications affect transcriptional initiation by altering the binding affinity between transcription factors and promoter regions or by regulating transcriptional elongation [2]. Unlike oncogenic mutations, epigenetic alterations can be reversible, making them potential therapeutic targets. Some DNA methyltransferase inhibitors and histone deacetylase inhibitors have been approved by the US Food and Drug Administration to treat certain hematologic malignancies [3,4]. However, single-agent epigenetic therapy has shown limited effects in solid tumors, including lung cancer [5]. Recently, histone methyltransferase and demethylase inhibitors targeting histone methylation have been reported [6]. More than 50 lysine methyltransferases and demethylases have been identified to date, and preliminary in vitro data targeting histone modifying enzymes have shown that they have promising antitumor activity in lung cancer cells [7,8].

Metformin, a first-line oral hypoglycemic agent for the treatment of type 2 diabetes mellitus, has been reported to exert antitumor properties in numerous types of cancer, including NSCLC. Mounting evidences from in vitro and in vivo studies have suggested the molecular mechanisms that underlie the antitumor effects of metformin [9]. Molecular properties such as the inhibition of reactive oxygen species and the AKT serine/threonine kinase/mechanistic target of rapamycin kinase (AKT/mTOR) pathway, and the activation of the AMP-activated protein kinase (AMPK) have suggested its potential as an antitumor agent. Recent studies also support the notion that metformin is likely to exert its anticancer activity by targeting epigenetic changes such as histone modifications. Metformin activates (AMPK) and inhibits histone H2B monoubiquitination in T47D breast cancer cells [10]. It also targets histone demethylase lysine demethylase 6A/Utx histone demethylase (KDM6A/UTX) and elevates global levels of H3K27me3 in the tumor tissues of mice bearing highly aggressive breast cancer xenografts [11]. The elevation of H3K27me3 via enhancer of zeste 2 polycomb repressive complex 2 subunit (EZH2) overexpression abrogated the effect that metformin had on cell proliferation and apoptosis in an ovarian cancer cell line, SKOV3 and ES2 cells [12].

Recently, we reported that metformin inhibits the proliferation of lung cancer cells by blocking cell cycle progression in the G1 phase through the downregulation of cyclins, cyclin dependent kinases (CDKs), and E2F transcription factors (E2Fs) [13]. However, the mechanisms by which metformin downregulates cell cycle regulators are not fully understood. To understand possible mechanisms underlying the downregulation of cell cycle regulatory genes by metformin, we analyzed histone H3 methylation, DNA methylation, and chromatin accessibility at the promoter regions of cell cycle regulatory genes in lung cancer cells.

## 2. Materials and Methods

### 2.1. Cell Culture

The H1299 and A549 lung cancer cell lines were obtained from the American Type Culture Collection. Those cell lines have been authenticated using short tandem repeat profiling in Samsung Medical Center for three years. The cells were cultured in RPMI 1640 medium (Lonza, Allendale, NJ, USA) supplemented with 10% fetal bovine serum, 100 U/mL penicillin, and 100 μg/mL streptomycin at 37 °C in a humidified atmosphere containing 5% CO_2_. The cells were treated with 5 mM metformin (D150959, Sigma-Aldrich, St. Louis, MO, USA) for 72 h. Five mM is the supraphysiological concentration of metformin. Metformin-induced growth inhibition was minimal at low concentrations (0.25 mM, 0.5 mM, and 1 mM) in our previous study [13]. Metformin clearly affected cell proliferation and cell cycle at high doses. In this study, the effect of metformin on histone modifications was not analyzed at lower concentrations since this study aimed at understanding metformin-induced histone modifications at the same concentration as 5 mM metformin that induced cell cycle arrest in the previous study. All experiments were performed with mycoplasma-free cells.

### 2.2. Global Histone H3 Modification Assay

Global histone H3 modifications (phosphorylation, methylation, and acetylation) were detected using an EpiQuik Histone H3 Modification Multiplex Assay Kit (Catalog No. P-3100-96, Epigentek, Farmingdale, NY, USA). Briefly, total core histones were extracted with an EpiQuik Total Histone Extraction Kit (Epigentek, Farmingdale, NY, USA). The isolated histones were added to wells coated with antibodies specific for 21 different histone H3 modifications. The captured histones were detected with a detection antibody, followed by a color development reagent. Absorbance intensity was measured at 450 nm with a microplate reader. The amount of a particular histone modification was calculated as a percentage of the total H3 signal.

### 2.3. ChIP-Seq Assay

Chromatin immunoprecipitation (ChIP) was performed with a SimpleChIP Enzymatic Chromatin IP Kit (Magnetic Beads) (#9003, Cell Signaling Technology, Danvers, MA, USA) according to the manufacturer’s instructions. Briefly, cells were cross-linked with 1% formaldehyde (Sigma-Aldrich) and lysed. The cross-linked chromatin was digested with micrococcal nuclease to a length of 150–900 bp of DNA/protein fragments. The fragments were immunoprecipitated with antibodies against H3K4me3 (C42D8, Cell Signaling Technology, Danvers, MA, USA), H3K27me3 (C36B11, Cell Signaling Technology), or normal rabbit IgG with protein G magnetic beads. The protein–DNA cross-links were reversed, and purified DNA was then subjected to next-generation sequencing (NovaSeq 6000 System, Illumina, San Diego, CA, USA). Sequence quality was checked using FastQC (version; 0.11.7), and reads were trimmed to remove low quality and adapter sequences using Trimmomatic software (version 0.38) [14]. Reads over Phred quality score of 20 were accepted as good quality. Adapter sequences and bases with base quality lower than three from the ends were removed. Also using the sliding window method, bases of reads that were not qualified for window size 4 and mean quality 15 were trimmed. Subsequently, reads with length shorter than 36 bp were dropped. After the read trimming, the sequence library was mapped to reference genome UCSC hg19 using the Bowtie1 program (version; 1.1.2). Peaks were identified using the MACS2 algorithm (version; 2.1.1) with following conditions: band width = 300; model fold = (10, 100) q-value cutoff ≤ 0.05; range for calculating regional lambda (1000 bps and 10,000 bps); broad region calling (off); paired-end mode (on); and searching for subpeak summits (on). Comparisons between paired samples were performed in the MAnorm program, and the *M*, *A*, and *p* values were calculated [15]. The *M*-value indicates the fold change in normalized read densities, the *A*-value denotes the average signal strength of the normalized read densities, and the *p*-value shows the significance of read intensity differences between two samples. Regions with *p*-values < 0.001 (−log10 *p*-value greater than 3) were considered statistically significant.

### 2.4. RNA-Seq Assay

Total RNA was isolated from cultured H1299 cells using a TruSeq Stranded mRNA LT Sample Prep Kit (Illumina, San Diego, CA, USA). The RNA was broken into short fragments after removing genomic DNA contamination with DNase I. The RNA fragments were reverse-transcribed into cDNA, ligated to adaptors, amplified by PCR, and sequenced using a next-generation sequencing device (NovaSeq 6000 System, Illumina, San Diego, CA, USA). The quality of the raw sequence data was checked using the FastQC program (version; 0.11.7); low-quality reads and PCR duplicates were removed using Trimmomatic 0.38. Quality filtering of all the RNA-seq raw data was performed using Trimmomatic software (version 0.38) by applying the same criteria used for trimming of Chip-seq data. Trimmed sequences were aligned to the reference transcripts using the HISAT2 program. The number of mapped reads was 83,118,285 and 73,808,022 for cells treated with metformin and untreated controls, respectively. Finally, the expression level of each transcript was estimated by counting the reads mapped to the transcripts. The read count was normalized into fragments per kilobase of transcript per million mapped reads value, and reads with a value of zero were excluded. Of the 27,685 genes, 10,654 were removed, and the remaining 17,031 genes were further analyzed. Significantly up- or downregulated genes were identified using a cutoff value of |FC| ≥ 1.5.

### 2.5. Gene-Set Enrichment Analysis

A Gene Ontology search and Gene Set Enrichment Analysis were performed using the Database for Annotation, Visualization, and Integrated Discovery (DAVID) and a Kyoto Encyclopedia of Genes and Genomes (KEGG) pathway analysis [16,17].

### 2.6. DNA Methylation Analysis

Genome-wide DNA methylation was analyzed using the SureSelect Methyl-Seq Target Enrichment System (Agilent Technologies, Santa Clara, CA, USA) according to the manufacturer’s protocol. Genomic DNA was isolated from cultured H1299 cells and randomly sheared. Library DNA was hybridized to biotinylated RNA probes (SureSelect Methyl-Seq Capture Library, Agilent Technologies) and recovered with streptavidin beads. Eluted DNA was treated with sodium bisulfite, amplified by PCR, and sequenced on an Illumina Genome Analyzer. Mapping of the sequenced reads was performed with the Bowtie2 program (version; 2.1.0). After duplicate removal using Bismark software [18], the number of mapped reads from cells treated with metformin and untreated controls was 47,803,761 and 54,906,440, respectively. Differentially methylated regions were defined as regions with more than five consecutive methylated CpGs and a ratio of observed to expected CpG of greater than or equal to 0.6. Regions with a *p*-value < 0.001 (Wilcoxon rank sum test) and a fold change |FC| ≥ 1.5 were considered significant.

### 2.7. ATAC-Seq Assay

The assay for transposase-accessible chromatin using sequencing (ATAC-seq) was performed by Active Motif (Carlsbad, CA, USA) as previously described [19]. Peaks were identified using the MACS2 algorithm at a cutoff of *p*-value < 1 × 10^−7^ with the non-model option. Significantly different regions were generated by applying the cutoffs MaxTags ≥ 100 and log2 ratio > 1 or < −1.

### 2.8. Quantitative Real-Time PCR (qRT- PCR)

Total RNA was isolated using a PureLink RNA Mini Kit (Invitrogen, Carlsbad, CA, USA) and reverse-transcribed using a SuperScript VILO cDNA Synthesis Kit (Invitrogen, Carlsbad, CA, USA). qRT-PCR was performed with SYBR green dye (4385614, Applied Biosystems, Foster City, CA, USA) under the following conditions: initial denaturation for 5 min at 95 °C, followed by 40 cycles of 5 s at 95 °C and 30 s at 60 °C. The PCR primer sequences are listed in Table S1. 

### 2.9. Western Blot Analysis

Total protein was extracted from cultured cells using lysis buffer containing a protease inhibitor cocktail (Roche Applied Science, Indianapolis, IN, USA). The lysates were heated for 5 min at 95 °C, loaded on 10% sodium dodecyl sulfate-polyacrylamide gels, and transferred to a PVDF membrane (Immobilon-*p*, Millipore, Medford, MA, USA). After blocking with a 3% solution of fetal bovine serum, the membranes were probed with the antibodies listed in Table S2. The membranes were then incubated with horseradish peroxidase-conjugated secondary antibodies (Cell Signaling Technology, Danvers, MA, USA) and visualized with an Immun-Star Western Kit (Bio-Rad, Hercules, CA, USA).

### 2.10. Small Interfering RNA (siRNA)-Mediated Gene Silencing

To knock down target genes, cells were transiently transfected with 40 nM gene-specific siRNA (BioNeer, DaeJeon, Korea) or non-targeting siRNA (BioNeer, DaeJeon, Korea) as a negative control. The siRNA sequences or their commercial IDs are listed in Table S3. Lipofectamine 2000 (Invitrogen, Carlsbad, CA, USA) was used to transfect the siRNA into cells according to the manufacturer’s protocol. At 48 h post-transfection, gene expression was measured using quantitative real-time PCR or western blot analyses.

### 2.11. Cell Viability Assay

Cells were cultured in 6-well plates (10,000 cells/well) and transfected with specific siRNA at a concentration of 40 nM. Cell viability was measured using a WST-8 [2-(2-methoxy-4-nitrophenyl)-3-(4-nitrophenyl)-5-(2,4-disulfophenyl)-2H tetrazolium, monosodium salt] assay kit (Biomax, Seoul, Korea) according to the manufacturer’s protocol. Absorbance was measured at 450 nm using a microplate reader (Bio-Rad, Hercules, CA, USA).

### 2.12. Statistical Analysis

Univariate analyses were performed using the Student’s *t*-test or Wilcoxon rank sum test and Pearson’s chi-square test or Fisher’s exact test for continuous and categorical variables, respectively. Linear relationships between two continuous variables were analyzed using the Pearson correlation coefficient. The effect of lysine methyltransferase 2B (MLL2) overexpression on survival was estimated using Kaplan–Meier survival curves, and the difference between the survival times of two independent groups was evaluated by the log-rank test. A Cox proportional hazards analysis was conducted to estimate the hazard ratios for MLL2 overexpression on survival after controlling for potential confounding factors. Statistical analyses were conducted using R software (version 3.6.1).

## 3. Results

### 3.1. Metformin Reduces Histone H3K4me3 at the Promoter Regions of Positive Cell Cycle Regulatory Genes in Lung Cancer Cells

**Figure 1:**
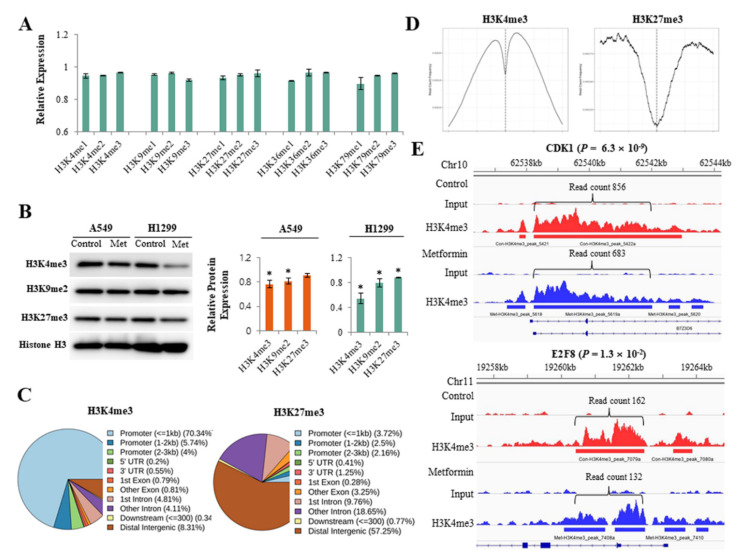
Effects of metformin on histone H3 modifications. H1299 and A549 cells were treated with 5 mM metformin or left untreated as controls. (**A**) Global alterations in histone H3 methylations were analyzed using an enzyme-linked immunosorbent assay. The *y*-axis indicates the amounts of proteins in metformin-treated cells relative to untreated cells. Error bars indicate the standard deviation (*n* = 3). (**B**) Protein levels of H3K4me3, H3K9me2, and H3K27me3 were analyzed by western blotting. The bar graphs show the expression of three proteins in cells treated with 5 mM metformin relative to untreated cells. Error bars indicate the standard deviation (*n* = 3, * *p* < 0.05). The uncropped blots of (**B**) were shown in Figure S1. (**C**) The results from the ChIP-seq analysis show DNA regions in which H3K4me3 or H3K27me3 modification were enriched in response to metformin. (**D**) The read count frequency of H3K4me3 or H3K27me3 within the transcription start site (TSS) ± 3 kb is shown. (**E**) The images show ChIP-seq peaks of two representative genes whose H3K4m3 was reduced by metformin. Peaks indicate read count frequency.

Global alterations of histone H3 methylation were analyzed using an enzyme-linked immunosorbent assay. Metformin inhibited various histone H3 methylation at lysine 4, 9, 27, 36, and 79, but the effect was minimal (Figure 1A). To more clearly evaluate the effect of metformin on histone H3 methylation, we analyzed the global levels of H3K4me3, H3K9me2, and H3K27me3, which are all important in lung cancer pathogenesis, using western blotting (Figure 1B). Metformin reduced H3K4me3 and H3K9me2 in both A549 and H1299 cells and H3K27me3 in H1299 cells. ChIP-seq was performed to find the DNA regions in which metformin induced alterations in H3K4me3 and H3K27me3. The ChIP-seq data are available at the Gene Expression Omnibus (GEO) with the ID: GSE141053. More than 80% of H3K4me3 was detected at gene promoters, whereas less than 10% of H3K27me3 was observed at promoters. Most (70%) of H3K4me3 was enriched at a transcription start site (TSS) ± 1 kb, and H3K27me3 was largely located outside of gene promoters (Figure 1C,D). Metformin significantly reduced H3K4me3 level at the promoter regions of 1113 genes and increased the level at the promoter regions of 1492 genes. The Gene Set Enrichment Analysis revealed that H3K4me3 levels decreased at the promoters of 88 cell cycle regulatory genes in response to metformin in lung cancer cells (Table S4). At the promoter regions of cyclin dependent kinase inhibitor 1A (CDKN1A/p21) and cyclin dependent kinase inhibitor 1B (CDKN1B/p27), H3K4me3 was upregulated in response to metformin, but the difference was statistically significant only in CDKN1A (Table S5). Representative ChIP-seq images of H3K4me3 reduction are shown in Figure 1E.

### 3.2. H3K4me3 Reduction at the Promoter Regions of Positive Cell Cycle Regulatory Genes Is Associated with mRNA Downregulation

**Figure 2:**
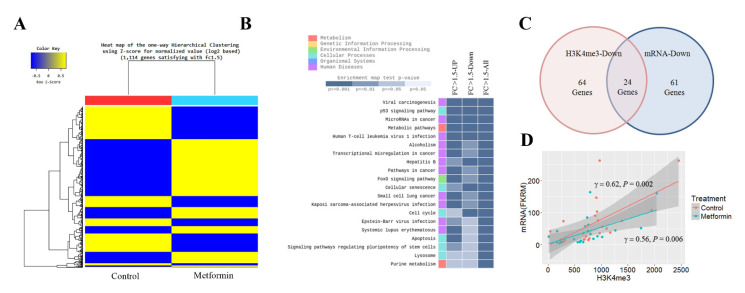
Effects of metformin on mRNA expression. The mRNA levels in H1299 cells treated with 5 mM metformin or left untreated as controls were analyzed by RNA-seq. (**A**) Heatmap shows one-way hierarchical clustering to stratify differentially expressed genes in treated and untreated cells. (**B**) Genes significantly up- or downregulated in response to metformin were identified using the cutoff value |FC| ≥ 1.5. The top 20 KEGG (Kyoto Encyclopedia of Genes and Genomes) pathway terms for genes with more than a 1.5-fold change in their mRNA levels are shown. (**C**) The number of cell cycle genes with reduced H3K4me3 at the promoter regions or downregulated mRNA in cells treated with metformin is shown. (**D**) The correlation between H3K4me3 and mRNA levels was analyzed for twenty-four genes with reduced H3K4me3 and mRNA.

To identify genes whose transcription was significantly altered by metformin, we performed RNA-seq in H1299 cells. The RNA-seq data are available at the GEO with the ID: GSE141052. The expression levels of 1114 genes were altered by 1.5 times or more in cells treated with metformin: the mRNA levels of 499 genes were downregulated, and those of 615 genes were upregulated (Figure 2A). The KEGG enrichment analysis showed that the genes significantly up- or downregulated in response to metformin were involved in the cell cycle, apoptosis, cellular senescence, and p53 signaling pathways (Figure 2B). Positive cell cycle regulators such as cyclin A2, cyclin E2, cyclin dependent kinase 1 (CDK1), E2F transcription factor 2, 6 and 8 (E2F2, E2F6, and E2F8) were downregulated, whereas negative cell cycle regulators such as cyclin dependent kinase inhibitor 2B (CDKN2B/p15), CDKN1A/p21, DNA damage inducible transcript 4 (DDIT4), and growth arrest and DNA damage inducible alpha (GADD45) were upregulated. Eighty-five downregulated genes involved in the cell cycle are listed in Supplementary Table S6. Of the 85 genes, 24 genes (28%) including marker of proliferation Ki-67 (MKI67), E2F8, CDK1, cell division cycle 7 (CDC7), and ubiquitin like with PHD and ring finger domains 1 (UHRF1) were showed reduction of H3K4me3 at their promoter regions (Figure 2C and Supplementary Table S7), and H3K4me3 levels were positively related to these mRNA levels (Figure 2D).

### 3.3. Effect of Metformin on Histone H3 Methyltransferase Is Independent of AMPK

**Figure 3:**
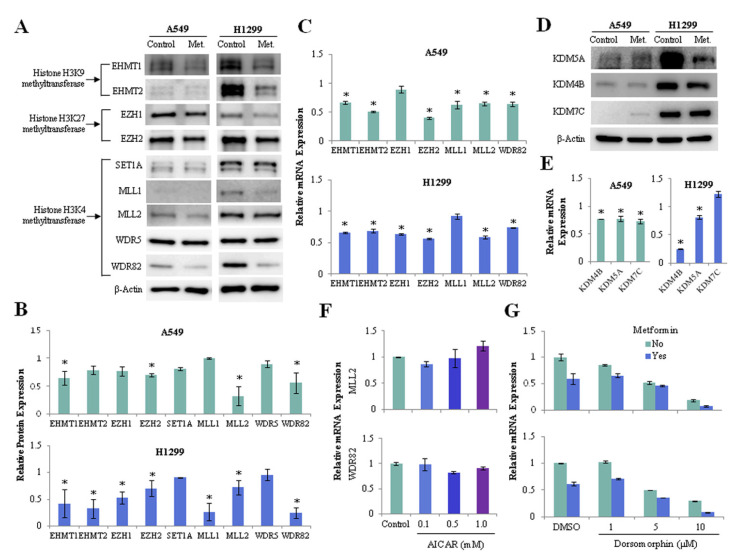
Effects of metformin on the expression of histone H3 methyltransferase and demethylase. H1299 and A549 cells were treated with 5mM metformin or left untreated as controls. (**A**) Protein levels of the H3K4 methyltransferases SET1A, MLL1, MLL2, WDR5, and WDR82; the H3K9 methyltransferases EHMT1 and EHMT2; and the H3K27 methyltransferases EZH1 and EZH2 were analyzed using western blotting. The uncropped blots of (**A**) were shown in Figure S2. (**B**) The expression levels of the methyltransferases in metformin-treated cells relative to control cells are shown. (**C**) mRNA levels of the methyltransferases were measured by qRT-PCR. The *y*-axis indicates the fold change in mRNA levels in metformin-treated cells compared with those in untreated control cells. (**D**) The protein levels of the H3K4 demethylase KDM5A and the H3K9 demethylases KDM4B and KDM7C were analyzed using western blotting. The expression levels of the demethylases in metformin-treated cells relative to control cells were shown in Figure S3A, and the uncropped blots of (**D**) were shown in Figure S3B. (**E**) The mRNA levels of three demethylases in metformin-treated cells relative to those in untreated cells. (**F**,**G**) H1299 cells were incubated with the AM*P*K activator AICAR or the AMPK inhibitor dorsomorphin (Dorso) with or without 5 mM metformin. The mRNA levels of MLL2 and WDR82 were measured by qRT-PCR. The error bars in all bar graphs indicate the standard deviation (*n* = 3, * *p* < 0.05).

We investigated whether metformin regulates the expression of histone H3 methyltransferase and demethylase in lung cancer cells. Metformin suppressed the protein (Figure 3A,B) and mRNA (Figure 3C) expressions of euchromatic histone lysine methyltransferase 1, 2 (EHMT1, EHMT2), enhancer of zeste 1, 2 polycomb repressive complex 2 subunit (EZH1, EZH2), lysine methyltransferase 2A, 2B (MLL1, MLL2), and WD repeat domain 82 (WDR82) in lung cancer cells. Metformin also reduced the expression of H3K4 demethylase lysine demethylase 5A (KDM5A/JARID1A) and H3K9 demethylase lysine demethylase 4B (KDM4B/JMJD2B) at the levels of protein (Figure 3D) and mRNA (Figure 3E) in A549 and H1299 cells. Because metformin is known to exert its antitumor action partly through AMPK activation, we analyzed whether its effect on the expression of histone H3 methyltransferases depended on AMPK. AMPK activation by 5-aminoimidazole-4-carboxamide ribonucleoside (AICAR) did not suppress mRNA expression of MLL2, WDR82 (Figure 3F), but inhibition of AMPK using dorsomorphin suppressed the expression of these genes (Figure 3G). Thus, the downregulation of histone H3 methyltransferases by metformin is independent of AMPK.

### 3.4. MLL2 Knockdown Significantly Reduces the Expression of H3K4me3 and Positive Cell Cycle Regulators

**Figure 4:**
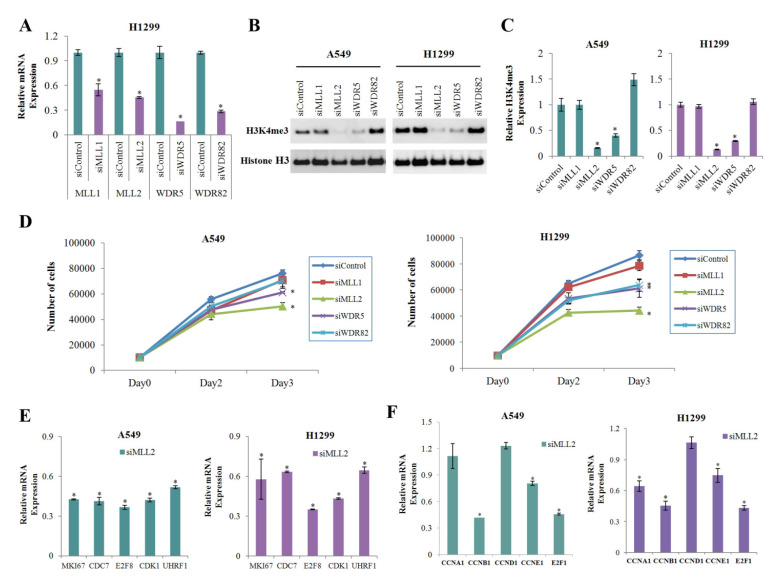
Effects of MLL2 on H3K4me3 and the mRNA levels of positive regulators of cell cycle. A549 and H1299 cells were transfected with siMLL1, siMLL2, siWDR5, siWDR82, and the off-target control siRNA (siControl). (**A**) The relative mRNA levels of the targeted genes were measured by qRT-PCR (*n* = 3, * *p* < 0.05). (**B**) The expression level of H3K4me3 was analyzed by western blotting. The uncropped blots of (**B**) were shown in Figure S4. (**C**) Levels of H3K4me3 in the siRNAs relative to the siControl. The western blot analysis was performed three times, and the average value was calculated. Error bars indicate the standard deviation (*n* = 3, * *p* < 0.05). (**D**) Cells were transfected with the indicated siRNAs, and the number of cells was calculated using the WST-8 assay on the zero, second, and third days after transfection (*n* = 4, * *p* < 0.05). (**E**,**F**) The mRNA levels of positive regulatory genes of cell cycle whose H3K4me3 levels were reduced significantly (**E**) or were not changed (**F**) in response to metformin were measured by qRT-PCR. Values are expressed as fold changes from siMLL2 to siControl (*n* = 3, * *p* < 0.05). The error bars in all bar graphs indicate the standard deviation (*n* = 3, * *p* < 0.05).

To find the H3K4 methyltransferase that was most significantly involved in the reduction of H3K4me3 in cells treated with metformin, we performed gene knockdown experiment on the H3K4 methyltransferases MLL1, MLL2, WD repeat domain 5 (WDR5), and WDR82 using respective siRNA. The knockdown efficiencies were confirmed by real-time PCR analysis (Figure 4A). Knockdown of MLL2 most significantly reduced global level of H3K4me3 (Figure 4B,C) and inhibited cell proliferation (Figure 4D) in both A549 and H1299 cells. The number of cells on the third day after siMLL2 transfection has decreased to 66% and 51% comparing with the siControl condition in A549 and H1299 cells, respectively (Figure 4D). MLL2 knockdown downregulated expression of the positive cell cycle regulators showed significant H3K4me3 reduction at their promoters in response to metformin (Figure 4E). Expressions of some other important cell cycle regulators such as cyclin B1 (CCNB1), cyclin E1 (CCNE1), and E2F1 were also dowregulated in siMLL2 cells (Figure 4F).

### 3.5. Metformin Does Not Significantly Change DNA Methylation or Chromatin Accessibility at the Promoter Regions of Positive Cell Cycle Regulatory Genes

**Figure 5:**
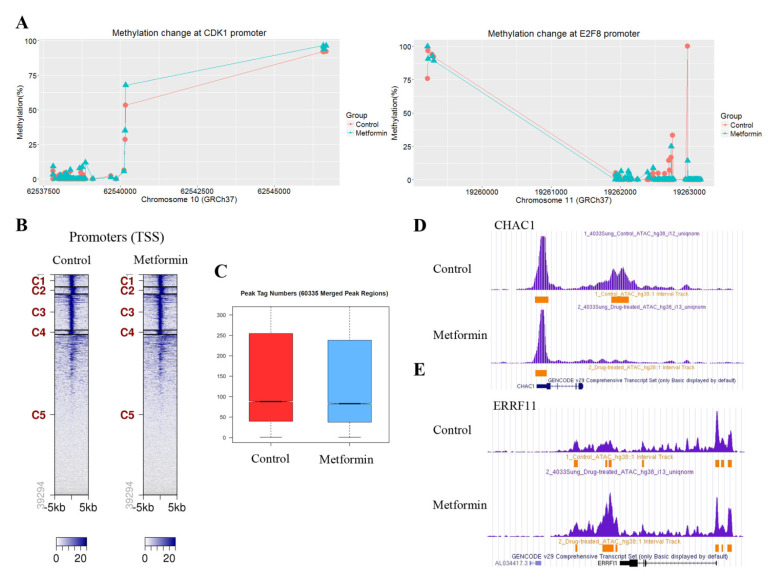
Effects of metformin on DNA methylation and chromatin accessibility. Alterations to DNA methylation and chromatin accessibility in H1299 cells treated with metformin or left untreated as controls were analyzed by the SureSelect Methyl-Seq Target System and ATAC-seq, respectively. (**A**) The methylation levels of CpGs at the promoter regions of CDK1 (left) and E2F8 (right) were compared between untreated and metformin-treated cells. (**B**) Heatmaps from the ATAC-seq analysis show the tag distribution in a 5-kb window in cells treated with metformin and left untreated. The promoter regions were grouped into five clusters by the bigWIG metrics program. (**C**) The peak tag number in the total merged peak regions was compared between cells treated and untreated with metformin. (**D**,**E**) The regions with significantly up- or downregulated chromatin accessibility in response to metformin were identified using the cutoff value |Log2 Ratio| ≥ 1. The images show that regions with severely downregulated (**D**) or upregulated (**E**) chromatin accessibility in response to metformin are located outside the promoter areas of the CHAC1 and ERRFI1 genes. Peaks indicate read count frequency.

To understand whether the observed changes in the mRNA levels of cell cycle regulatory genes in response to metformin are associated with DNA methylation, we analyzed the methylation levels of CpGs at the promoter regions of the genes using the SureSelect Methyl-Seq Target Enrichment System. Some regions at the genome level were found to be hyper- or hypomethylated, but most of the cell cycle regulatory genes did not show significantly altered DNA methylation like that seen in CDK1 and E2F8 (Figure 5A). ATAC-seq was performed to investigate whether the downregulation of positive cell cycle regulators in cells treated with metformin was associated with changes in chromatin accessibility. The ATAC-seq data are available at the GEO with ID: GSE141059. Chromatin accessibility in cells treated with metformin decreased in 159 regions and increased in 124 regions. Most regions with changes in chromatin accessibility were outside of promoter regions defined as TSS ± 3kb. Tag distribution (Figure 5B) in the promoter regions and the peak tag number in the total merged peak (Figure 5C) were found to be similar. The regions showing the greatest increase and decrease in chromatin accessibility were located 4667bp downstream of ChaC glutathione specific gamma-glutamylcyclotransferase 1 (CHAC1) (Figure 5D) and 15,111 bp downstream of ERBB receptor feedback inhibitor 1 (ERRFI1) (Figure 5E), respectively. We identified 54 genes whose chromatin accessibility was significantly changed at the promoter regions, but most of those genes were not related to cell cycle regulation (Table S8). We further analyzed the chromatin accessibility at the promoter regions of the approximately 2500 genes where the H3K4me3 level was significantly altered by metformin. The average change (log2Ratio) of chromatin accessibility in the 1113 genes showing H3K4me3 downregulation by metformin was a little greater than the change in the 1492 gens showing H3K4me3 upregulation (−0.11 vs. −0.07), but the difference was not statistically significant (*p* = 0.09; Student’s *t*-test). Based on these observations, it is reasonable to speculate that H3K4me3 alterations by metformin at promoter regions may not be associated with chromatin accessibility.

### 3.6. Clinicopathological Characteristics of MLL2 Overexpression in Primary NSCLC

**Figure 6:**
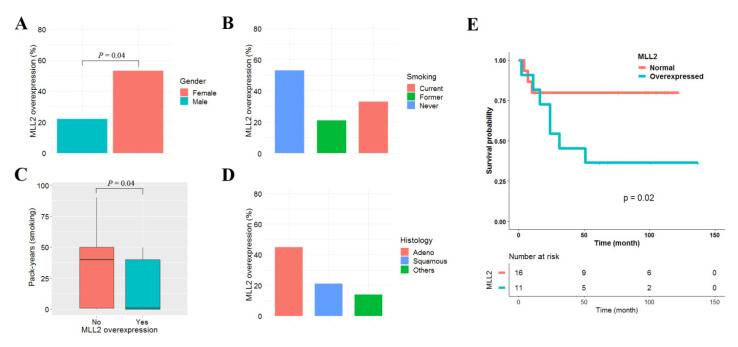
Effect of MLL2 overexpression on recurrence-free survival in lung adenocarcinoma. Clinicopathological significance of MLL2 overexpression in 42 primary NSCLCs. MLL2 overexpression was compared according to sex (**A**), smoking status (**B**), pack-years (**C**), and histology (**D**). The effect of MLL2 overexpression on the recurrence-free survival of 27 patients with adenocarcinoma was analyzed using Kaplan–Meier survival curves (**E**). The *p*-value was calculated using the log-rank test.

We used previously reported data to analyze the clinicopathological significance of MLL2 overexpression in 42 primary NSCLCs [20]. MLL2 was defined to be overexpressed when its mRNA level was ≥1.5 fold higher in tumor tissues than in matched normal tissue. MLL2 overexpression was found in 14 (32%) of the 42 NSCLCs tested, and was not associated with patient age, pathologic stage, or differentiation (data not shown). However, MLL2 overexpression was found to be highly prevalent in females (*p* = 0.04, Figure 6A). Although MLL2 overexpression was not statistically associated with smoking status (*p* = 0.34; Figure 6B), it tended to occur more frequently in never-smokers: the number of pack-years for patients with and without MLL2 overexpression was 17 vs. 21 and 34 vs. 28, respectively *(p* = 0.04; Figure 6C). MLL2 overexpression was also found in adenocarcinoma at a higher prevalence than in squamous cell carcinoma, though that difference was not statistically significant (*p* = 0.27; Figure 6D). The effect of MLL2 overexpression on patient survival was analyzed in the 42 NSCLCs. The median follow-up period for the patients was 5.3 years. MLL2 overexpression was significantly associated with poor recurrence-free survival in adenocarcinoma (*p* = 0.02; Figure 6E) but not in squamous cell carcinoma (*p* = 0.87). The five-year recurrence-free survival rate of 27 adenocarcinoma patients with and without MLL2 overexpression was 34% and 78%, respectively. A Cox proportional hazards analysis showed that the recurrence-free survival of adenocarcinoma patients with MLL2 overexpression was approximately 1.32 (95% CI = 1.12–4.57; *p* = 0.01) times poorer than in patients without MLL2 overexpression after controlling for sex and pathologic stage (Table S9).

## 4. Discussion

A nucleosome is composed of genomic DNA wrapped around an octamer that contains two copies of each of the histone proteins H2A, H2B, H3, and H4. Five lysines in histone H3 (K4, K9, K27, K36, K79) and a lysine in histone H4 (K20) are known to be modulated by methylation [21]. H3K4me3 is present at the promoters of active genes and leads to low nucleosome density at those promoters, and H3K36me3 is associated with elongating RNA polymerase II and occurs in the bodies of active genes. H3K9me2/3 is associated with constitutive heterochromatin formation at promoters and strongly associated with gene repression. H3K27me3 and H4K20me3 are also tightly associated with heterochromatin as clear markers of gene repression [21]. In this study, we investigated the effect of metformin on epigenetic alterations (histone H3 methylation, DNA methylation, and chromatin accessibility) in lung cancer cells. Although the effects of metformin on DNA methylation and chromatin accessibility were minimal, metformin downregulated H3K4me3, especially at the promoter regions of positive regulatory genes of cell cycle.

H3K4me3 is found in ∼75% of all human active gene promoters in several cell types [22], and the presence of this mark at the promoters of protein coding genes is known to correlate with an active state of gene expression [23]. Many studies have reported the molecular mechanisms that underlie the regulation of gene expression by H3K4me3. H3K4me3 is known to positively regulate gene expression through chromatin remodeling. Recruitment of the chromatin remodeling complex NURF (Nucleosome Remodeling Factor) by H3K4me3 allows the transcription factors more access to DNA. Transcriptional pre-initiation complex formation is facilitated by recruiting transcription factor II D (TFIID) via the TATA binding protein associated factor 3 (TAF3) [24]. TAF3, as a component of TFIID, interacts with H3K4me3 and provides the mechanism by which TFIID is recruited to the promoter. TAF3 binding sites are significantly enriched at cell cycle regulatory genes [25]. In contrast, the loss of H3K4me3 inhibits transcription by reducing TFIID binding to promoters [25,26]. In this study, approximately 80% of H3K4me3 was found to be enriched in gene promoters, and 24 (27%) of 88 cell cycle genes with downregulated H3K4me3 in response to metformin also showed reduced mRNA levels. Based on these observations, it is likely that metformin affects the expression of cell cycle regulatory genes at least partially through the reduction of H3K4me3 at their promoter regions.

AMPK activation is known to be one of the mechanisms underlying the antineoplastic effect of metformin. Metformin can inhibit cancer cell growth by suppressing mammalian target of rapamycin complex 1 (mTORC1) in AMPK-dependent and AMPK-independent pathways [27]. Several groups have reported that metformin could inhibit mTORC1 activation in the absence of AMPK. Metformin inhibited mTORC1 in a GTPase-dependent manner in HEK293T cells and did not have a pronounced effect on the energy status of AMPK dKO MEFs in the absence of AMPK [28]. AMPK inhibition had a minor impact on the metformin effect, which induced mTOR inhibition and cell cycle arrest through REDD1 in prostate cancer cells [29]. In addition, metformin suppressed the proliferation of glioma cells through PRAS40-mediated mTOR inhibition independent of AMPK [30]. In this study, we also found that the effect of metformin on H3K4 methyltransferase expression did not depend on AMPK activation (Figure 3F,G). Accordingly, the ability of metformin to downregulate histone H3K4 methyltransferase in lung cancer cells could be independent of AMPK activation despite the contribution of the AMPK/mTOR signaling pathway to metformin-induced anticancer therapy.

The transfer of methyl groups to lysine residues on histone H3K4 is known to be principally catalyzed by methyltransferase. MLL1 and MLL2 are members of the SET1/COMPASS complex and control the expression of actively transcribed genes by regulating H3K4me3 [31]. WDR82, as a core subunit of the SET1A complex, mediates recruitment of the SET1A histone methyltransferase complex to transcription start sites [32]. In this study, metformin significantly inhibited the expression of MLL1, MLL2, and WDR82 in lung cancer cells (Figure 3A). H3K4me3 was most significantly downregulated in A549 and H1299 cells treated with siMLL2 rather than siMLL1 or siWDR5 (Figure 4B,C), suggesting that MLL2 could function as a core factor regulating H3K4me3 in lung cancer cells. Several groups have reported that MLL2 plays an oncogenic role in human cancer. MLL2 overexpression compared with adjacent benign epithelium was reported in invasive carcinomas of the breast and colon [33]. MLL2 knockdown resulted in a significant decrease in the migration of esophageal squamous cell carcinoma cells. Clinically, a high level of MLL2 is significantly associated with early-stage ESCC lymph node metastasis [34]. Knockdown of MLL2 and KDM6A in NSCLC cell lines markedly inhibited the tumorigenic phenotype both in vitro and in vivo [35]. In this study, MLL2 knockdown also significantly inhibited the proliferation of lung cancer cells (Figure 4D) and downregulated positive cell cycle regulators (Figure 4E,F). These observations suggest that MLL2 might function as an oncogene in some kinds of human cancers, and may play an important role in reducing H3K4me3 at the promoter regions of positive cell cycle regulatory genes in response to metformin in lung cancer cells.

MLL2 is known to be involved in tumor progression and is associated with poor prognosis in a variety of cancers. MLL2 knockout suppressed cell cycle progression by inducing cell cycle arrest at the G1 stage in esophageal squamous cell carcinoma cells in vitro and inhibited cell migration [36]. High MLL2 expression predicts poor prognosis and promotes tumor progression by inducing the EMT in esophageal squamous cell carcinoma [36]. MLL2 maintains overall H3K4me2/me3 levels in MLL-AF9 leukemia cells, and deletion of MLL2 reduces the survival of those cells in vitro and in vivo [37]. In addition, MLL2 overexpression was negatively associated with patient survival in gastrointestinal diffuse large B-cell lymphoma [38]. Consistent with these previous results, we found that MLL2 overexpression was significantly associated with poor recurrence-free survival in lung adenocarcinoma.

The human lysine demethylase 5 (KDM5) family consists of four members designated KDM5A–D and specifically catalyzes the demethylation of H3K4. KDM5A, also known as retinoblastoma binding protein (RBP2), was initially identified as a binding partner of retinoblastoma protein, and its overexpression has been observed in cancers such as glioblastoma, gastric cancer [39], hepatocellular carcinoma, and lung cancer [40]. Increasing evidence suggests that KDM5A plays an oncogenic role in tumorigenesis and the progression of human cancer. KDM5A promotes small cell lung cancer tumorigenesis by repressing NOTCH signaling and sustaining ASLC1 expression [41]. Wang et al. reported that KDM5A downregulated the expression of E-cadherin and that KDM5A overexpression induced the epithelial–mesenchymal transition in NSCLC cells [42]. KDM5A promotes cell cycle progression by repressing p27 and activating cyclins D1 and E1 in lung cancer cells [43]. In lung cancer cells, KDM5A binds directly to the promoter regions of cyclin D1 and cyclin-dependent kinase inhibitor p27 (KIP1) and promotes G1-S progression by activating cyclins D1 and E1 and suppressing p27 (CDKN1B) [40]. KDM5A also promotes the angiogenesis of NSCLC cells by upregulating VEGF and activating HIF-1α via PI3K/Akt signaling [43]. KDM5A depletion enhanced the promoter activities of p16 (INK4A), p21 (CIP1), and p27 (KIP1) and upregulated H3K4me3 levels in gastric cancer cells [39]. In this study, metformin suppressed KDM5A expression and upregulated H3K4me3 at the promoter regions of p21 and p27 (Supplementary Table S5). Thus, in lung cancer cells, metformin might increase p21 and p27 expression by upregulating H3K4me3 at the promoter region of each gene through the downregulation of KDM5A.

This study was limited by several factors. First, the clinical significance of MLL2 expression was retrospectively studied in only a small number of samples. Those results need to be confirmed in a prospective study with a large number of patients. Second, the effect of metformin-induced downregulation of MLL2 on histone H3K4me3 was investigated, but the influence of metformin-induced downregulation of KDM5A was not analyzed in this study. Metformin-induced H3K4me3 alteration needs to be analyzed after double knockdown of MLL2 and KDM5A.

## 5. Conclusions

In conclusion, the present study suggests that metformin may suppress the expression of positive cell cycle regulatory genes by reducing H3K4me3 at their promoter regions through MLL2 downregulation in lung cancer cells. Additionally, MLL2 may be a therapeutic target for reducing recurrence after surgery in lung adenocarcinoma.

## Data Availability

The datasets analyzed during the current study are available at the Gene Expression Omnibus (https://www.ncbi.nlm.nih.gov/geo (accessed on 9 February 2021).) with the IDs (GSE141053 and GSE141052).

## Supplementary Materials

The following are available online at https://www.mdpi.com/2072-6694/13/4/739/s1, Figure S1: Uncropped blots of Figure 1B; Figure S2: Uncropped blots of Figure 3A; Figure S3: (A) The expression levels of the demethylases in metformin-treated cells relative to control cells are shown. (B) Uncropped blots of Figure 3D; Figure S4: Uncropped blots of Figure 4B; Table S1: Primer sequences for qRT-PCR; Table S2: List of antibodies used in western blot analysis; Table S3: List of siRNAs used in the study; Table S4: List of cell cycle genes whose H3K4me3 levels at the promoter regions were significantly reduced in response to metformin; Table S5: H3K4me3 alteration at the promoter regions of CDKN1A (p21) and CDKN1B (p27) in response to metformin; Table S6: List of cell cycle genes whose mRNA levels were downregulated by greater than or equal to 1.5 fold in response to metformin; Table S7: List of cell cycle genes in which both H3K4me3 and mRNA were downregulated in response to metformin; Table S8: List of genes whose chromatin accessibility was significantly changed at promoter regions in response to metformin; Table S9: Cox proportional hazards analysis of survival according to MLL2 expression in lung adenocarcinoma (*n* = 27).

## References

[1] Siegel R.L., Miller K.D., Jemal A. (2016). Cancer statistics, 2016. CA Cancer J. Clin..

[2] Baylin S.B., Jones P.A. (2011). A decade of exploring the cancer epigenome-biological and translational implications. Nat. Rev. Cancer.

[3] Kaminskas E., Farrell A., Abraham S., Baird A., Hsieh L.S., Lee S.L., Leighton J.K., Patel H., Rahman A., Sridhara R. (2005). Approval summary: Azacitidine for treatment of myelodysplastic syndrome subtypes. Clin. Cancer Res..

[4] Duvic M., Talpur R., Ni X., Zhang C., Hazarika P., Kelly C., Chiao J.H., Reilly J.F., Ricker J.L., Richon V.M. (2007). Phase 2 trial of oral vorinostat (suberoylanilide hydroxamic acid, SAHA) for refractory cutaneous T-cell lymphoma (CTCL). Blood.

[5] Azad N., Zahnow C.A., Rudin C.M., Baylin S.B. (2013). The future of epigenetic therapy in solid tumours—Lessons from the past. Nat. Rev. Clin. Oncol..

[6] Morera L., Lubbert M., Jung M. (2016). Targeting histone methyltransferases and demethylases in clinical trials for cancer therapy. Clin. Epigenetics.

[7] Baumert H.M., Metzger E., Fahrner M., George J., Thomas R.K., Schilling O., Schule R. (2020). Depletion of histone methyltransferase KMT9 inhibits lung cancer cell proliferation by inducing non-apoptotic cell death. Cancer Cell Int..

[8] Zhang K., Wang J., Yang L., Yuan Y.C., Tong T.R., Wu J., Yun X., Bonner M., Pangeni R., Liu Z. (2018). Targeting histone methyltransferase G9a inhibits growth and Wnt signaling pathway by epigenetically regulating HP1alpha and APC2 gene expression in non-small cell lung cancer. Mol. Cancer.

[9] Li C., Xue Y., Xi Y.R., Xie K. (2017). Progress in the application and mechanism of metformin in treating non-small cell lung cancer. Oncol. Lett..

[10] Du Y., Zheng H., Wang J., Ren Y., Li M., Gong C., Xu F., Yang C. (2014). Metformin inhibits histone H2B monoubiquitination and downstream gene transcription in human breast cancer cells. Oncol. Lett..

[11] Cuyas E., Verdura S., Llorach-Pares L., Fernandez-Arroyo S., Luciano-Mateo F., Cabre N., Stursa J., Werner L., Martin-Castillo B., Viollet B. (2018). Metformin directly targets the H3K27me3 demethylase KDM6A/UTX. Aging Cell.

[12] Tang G., Guo J., Zhu Y., Huang Z., Liu T., Cai J., Yu L., Wang Z. (2018). Metformin inhibits ovarian cancer via decreasing H3K27 trimethylation. Int. J. Oncol..

[13] Jin D.H., Kim Y., Lee B.B., Han J., Kim H.K., Shim Y.M., Kim D.H. (2017). Metformin induces cell cycle arrest at the G1 phase through E2F8 suppression in lung cancer cells. Oncotarget.

[14] Bolger A.M., Lohse M., Usadel B. (2014). Trimmomatic: A flexible trimmer for Illumina sequence data. Bioinformatics.

[15] Shao Z., Zhang Y., Yuan G.C., Orkin S.H., Waxman D.J. (2012). MAnorm: A robust model for quantitative comparison of ChIP-Seq data sets. Genome Biol..

[16] Da Huang W., Sherman B.T., Lempicki R.A. (2009). Systematic and integrative analysis of large gene lists using DAVID bioinformatics resources. Nat. Protoc..

[17] Kanehisa M., Goto S., Furumichi M., Tanabe M., Hirakawa M. (2010). KEGG for representation and analysis of molecular networks involving diseases and drugs. Nucleic Acids Res..

[18] Krueger F., Andrews S.R. (2011). Bismark: A flexible aligner and methylation caller for Bisulfite-Seq applications. Bioinformatics.

[19] Buenrostro J.D., Giresi P.G., Zaba L.C., Chang H.Y., Greenleaf W.J. (2013). Transposition of native chromatin for fast and sensitive epigenomic profiling of open chromatin, DNA-binding proteins and nucleosome position. Nat. Methods.

[20] Um S.W., Kim H.K., Kim Y., Lee B.B., Kim D., Han J., Kim H., Shim Y.M., Kim D.H. (2017). Bronchial biopsy specimen as a surrogate for DNA methylation analysis in inoperable lung cancer. Clin. Epigenetics.

[21] Zhao Z., Shilatifard A. (2019). Epigenetic modifications of histones in cancer. Genome Biol..

[22] Consortium E.P. (2012). An integrated encyclopedia of DNA elements in the human genome. Nature.

[23] Santos-Rosa H., Schneider R., Bannister A.J., Sherriff J., Bernstein B.E., Emre N.C., Schreiber S.L., Mellor J., Kouzarides T. (2002). Active genes are tri-methylated at K4 of histone H3. Nature.

[24] Lauberth S.M., Nakayama T., Wu X., Ferris A.L., Tang Z., Hughes S.H., Roeder R.G. (2013). H3K4me3 interactions with TAF3 regulate preinitiation complex assembly and selective gene activation. Cell.

[25] Cano-Rodriguez D., Gjaltema R.A., Jilderda L.J., Jellema P., Dokter-Fokkens J., Ruiters M.H., Rots M.G. (2016). Writing of H3K4Me3 overcomes epigenetic silencing in a sustained but context-dependent manner. Nat. Commun..

[26] Vermeulen M., Mulder K.W., Denissov S., Pijnappel W.W., van Schaik F.M., Varier R.A., Baltissen M.P., Stunnenberg H.G., Mann M., Timmers H.T. (2007). Selective anchoring of TFIID to nucleosomes by trimethylation of histone H3 lysine 4. Cell.

[27] Pernicova I., Korbonits M. (2014). Metformin—Mode of action and clinical implications for diabetes and cancer. Nat. Rev. Endocrinol..

[28] Kalender A., Selvaraj A., Kim S.Y., Gulati P., Brule S., Viollet B., Kemp B.E., Bardeesy N., Dennis P., Schlager J.J. (2010). Metformin, independent of AMPK, inhibits mTORC1 in a rag GTPase-dependent manner. Cell Metab..

[29] Ben Sahra I., Regazzetti C., Robert G., Laurent K., Le Marchand-Brustel Y., Auberger P., Tanti J.F., Giorgetti-Peraldi S., Bost F. (2011). Metformin, independent of AMPK, induces mTOR inhibition and cell-cycle arrest through REDD1. Cancer Res..

[30] Liu X., Chhipa R.R., Pooya S., Wortman M., Yachyshin S., Chow L.M., Kumar A., Zhou X., Sun Y., Quinn B. (2014). Discrete mechanisms of mTOR and cell cycle regulation by AMPK agonists independent of AMPK. Proc. Natl. Acad. Sci. USA.

[31] Shilatifard A. (2008). Molecular implementation and physiological roles for histone H3 lysine 4 (H3K4) methylation. Curr. Opin. Cell Biol..

[32] Wu M., Wang P.F., Lee J.S., Martin-Brown S., Florens L., Washburn M., Shilatifard A. (2008). Molecular regulation of H3K4 trimethylation by Wdr82, a component of human Set1/COMPASS. Mol. Cell Biol..

[33] Natarajan T.G., Kallakury B.V., Sheehan C.E., Bartlett M.B., Ganesan N., Preet A., Ross J.S., FitzGerald K.T. (2010). Epigenetic regulator MLL2 shows altered expression in cancer cell lines and tumors from human breast and colon. Cancer Cell Int..

[34] Li H., Li Q., Lian J., Chu Y., Fang K., Xu A., Chen T., Xu M. (2020). MLL2 promotes cancer cell lymph node metastasis by interacting with RelA and facilitating STC1 transcription. Cell Signal.

[35] Leng X., Wang J., An N., Wang X., Sun Y., Chen Z. (2020). Histone 3 lysine-27 demethylase KDM6A coordinates with KMT2B to play an oncogenic role in NSCLC by regulating H3K4me3. Oncogene.

[36] Abudureheman A., Ainiwaer J., Hou Z., Niyaz M., Turghun A., Hasim A., Zhang H., Lu X., Sheyhidin I. (2018). High MLL2 expression predicts poor prognosis and promotes tumor progression by inducing EMT in esophageal squamous cell carcinoma. J. Cancer Res. Clin. Oncol..

[37] Chen Y., Anastassiadis K., Kranz A., Stewart A.F., Arndt K., Waskow C., Yokoyama A., Jones K., Neff T., Lee Y. (2017). MLL2, Not MLL1, Plays a Major Role in Sustaining MLL-Rearranged Acute Myeloid Leukemia. Cancer Cell.

[38] Ye H., Lu L., Ge B., Gao S., Ma Y., Liang B., Yu K., Yang K. (2015). MLL2 protein is a prognostic marker for gastrointestinal diffuse large B-cell lymphoma. Int. J. Clin. Exp. Pathol..

[39] Zeng J., Ge Z., Wang L., Li Q., Wang N., Bjorkholm M., Jia J., Xu D. (2010). The histone demethylase RBP2 Is overexpressed in gastric cancer and its inhibition triggers senescence of cancer cells. Gastroenterology.

[40] Teng Y.C., Lee C.F., Li Y.S., Chen Y.R., Hsiao P.W., Chan M.Y., Lin F.M., Huang H.D., Chen Y.T., Jeng Y.M. (2013). Histone demethylase RBP2 promotes lung tumorigenesis and cancer metastasis. Cancer Res..

[41] Oser M.G., Sabet A.H., Gao W., Chakraborty A.A., Schinzel A.C., Jennings R.B., Fonseca R., Bonal D.M., Booker M.A., Flaifel A. (2019). The KDM5A/RBP2 histone demethylase represses NOTCH signaling to sustain neuroendocrine differentiation and promote small cell lung cancer tumorigenesis. Genes Dev..

[42] Wang S., Wang Y., Wu H., Hu L. (2013). RBP2 induces epithelial-mesenchymal transition in non-small cell lung cancer. PLoS ONE.

[43] Qi L., Zhu F., Li S.H., Si L.B., Hu L.K., Tian H. (2014). Retinoblastoma binding protein 2 (RBP2) promotes HIF-1alpha-VEGF-induced angiogenesis of non-small cell lung cancer via the Akt pathway. PLoS ONE.

