# Supplementary material for: Metformin Reduces Histone H3K4me3 at the Promoter Regions of Positive Cell Cycle Regulatory Genes in Lung Cancer Cells
Source: Cancers (Basel). 2021 Feb 10;13(4):739. doi: 10.3390/cancers13040739 (PMC7916663; doi:10.3390/cancers13040739)

Figure S1: Uncropped blots of Figure 1B

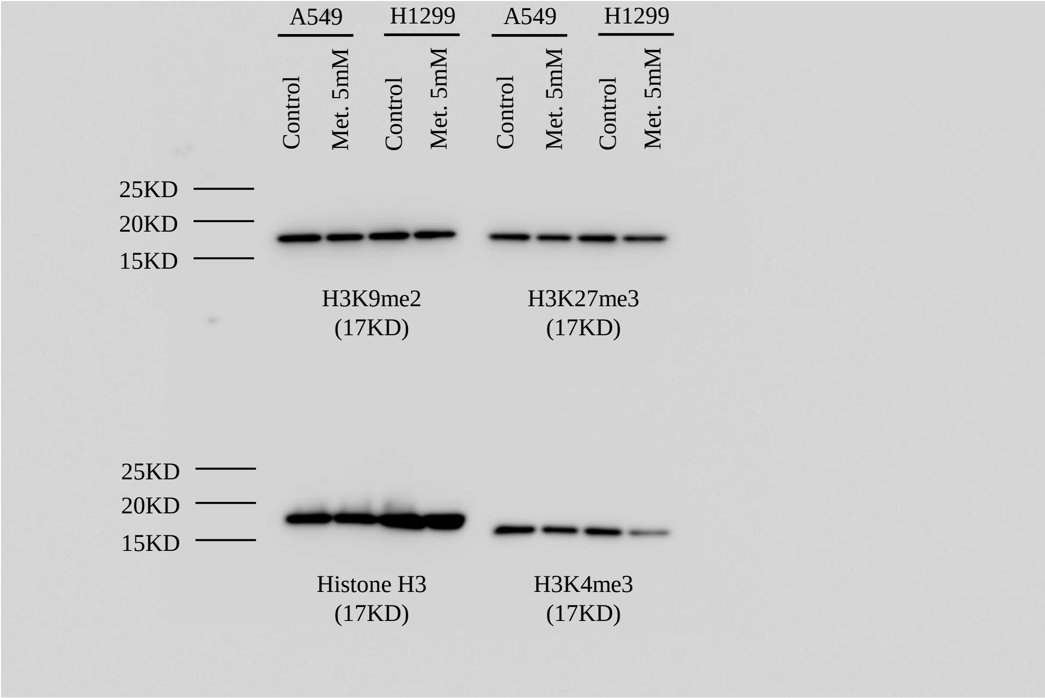

Figure S2: Uncropped blots of Figure 3A

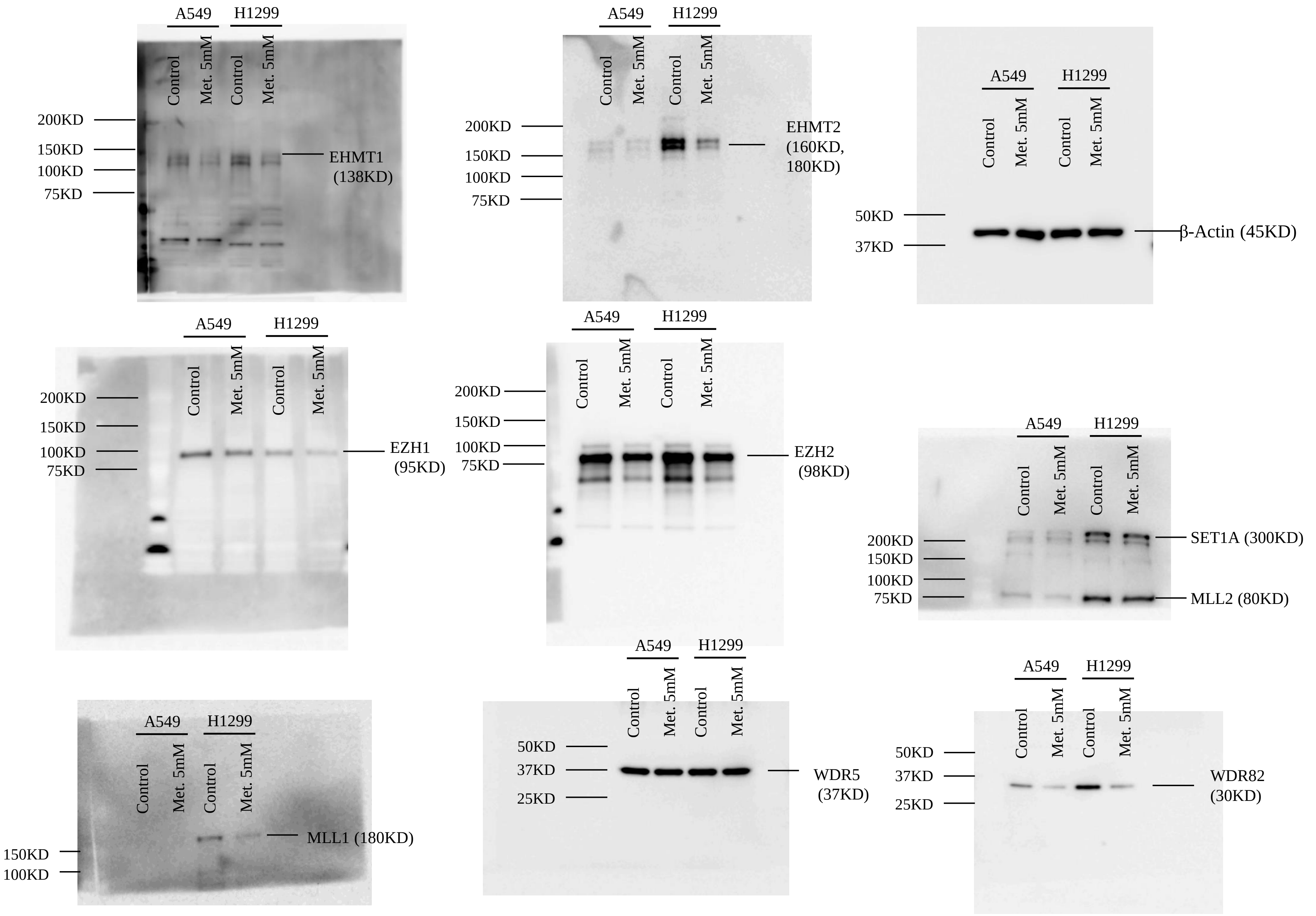

Figure S3: (A) The expression levels of the demethylases in metformin-treated cells relative to control cells are shown. **(B)** Uncropped blots of Figure 3D

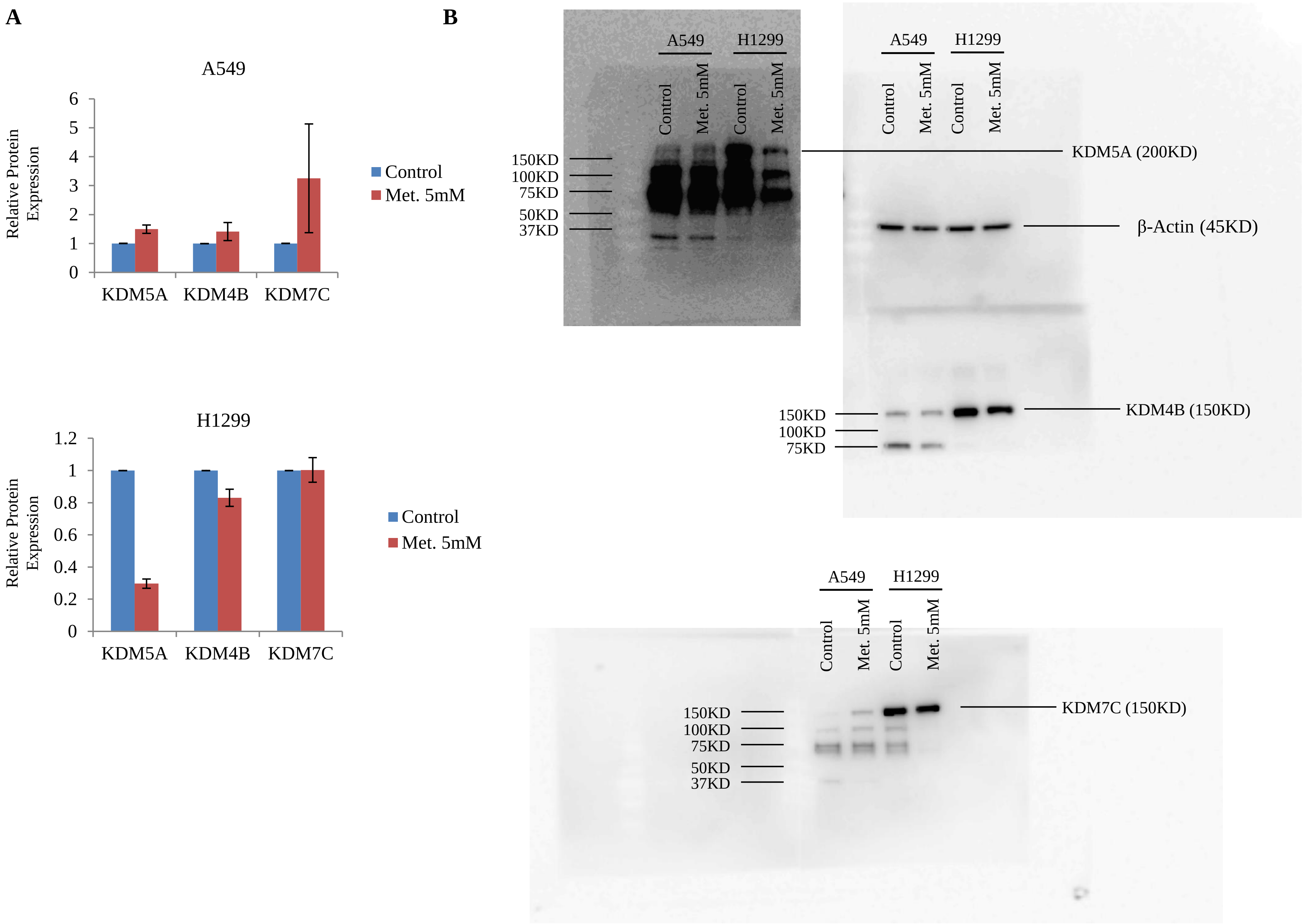

Figure S4: Uncropped blots of Figure 4B

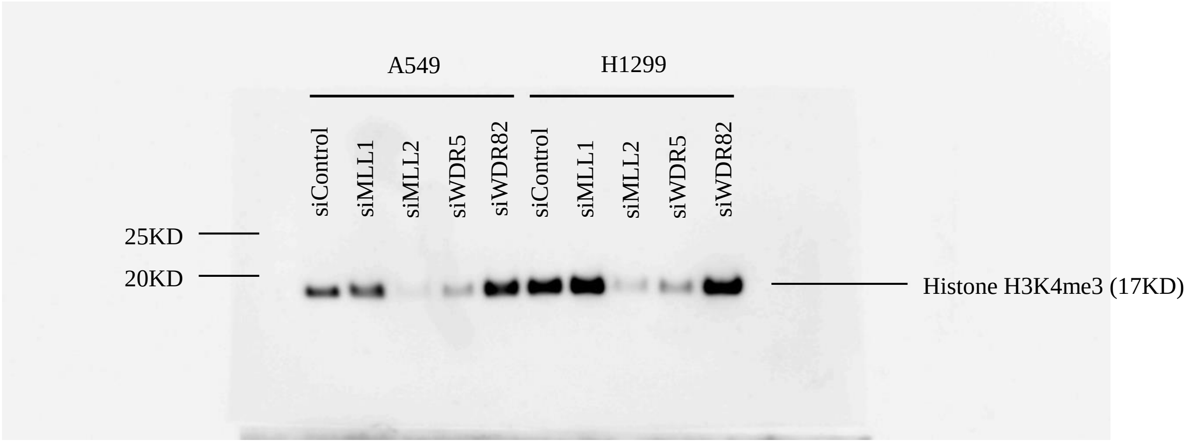

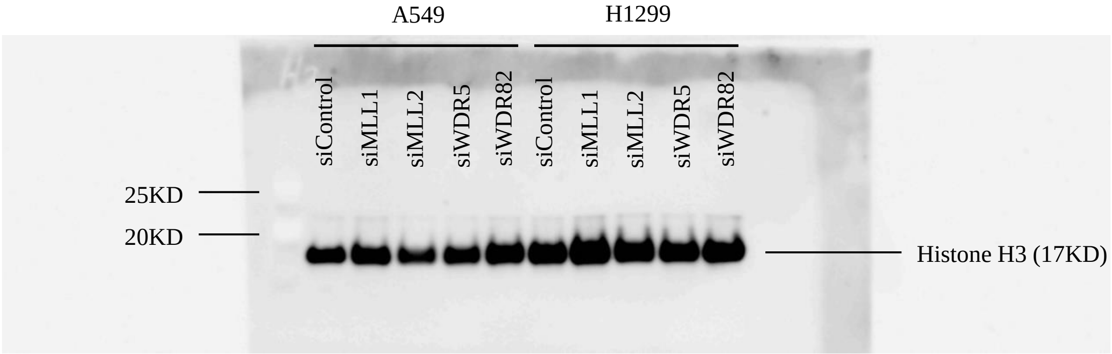

Supplement: Supplementary file 1 [file cancers-13-00739-s001.zip › Supplementary Figures.pdf]
