# Supplementary material for: Metformin Reduces Histone H3K4me3 at the Promoter Regions of Positive Cell Cycle Regulatory Genes in Lung Cancer Cells
Source: Cancers (Basel). 2021 Feb 10;13(4):739. doi: 10.3390/cancers13040739 (PMC7916663; doi:10.3390/cancers13040739)
Supplement: Supplementary file 1 [file cancers-13-00739-s001.zip › Supplementary Tables.pdf]

SUPPLEMENTARY TABLES

Supplementary Table S1. Primer sequences for qRT-PCR

Gene Name	Forward (5' → 3')	Reverse (5' → 3')
EHMT1	ggatcgccttcttcagcac	agctgaagagcttgcctttg
EHMT2	tgctgaggctgatgtgagag	tgccatagtaacgggcatct
EZH1	tgaatggagaccatcggatt	gagagcatcagcttggctgt
EZH2	tttttgccaagagagccatc	tcgatgccgacatacttcag
MLL1	gtcccacaaggtctccttca	cggtcagagccacttctagg
MLL2	ctcggtgttgactgacaagc	tccatgcggaacatatagca
WDR5	catctggaaccttcagacga	ttgggtgacaagctgttgag
WDR82	ttcatgacttttgccagtgc	gccgccctcactatacagaa
KDM4A	ccaagtgagatggacagacg	gtgagccatcctcaaactcc
KDM4B	tcaccagccacatctaccag	tcctccagggtgaagatgtc
KDM5A	cagcacagaactgccaaagg	ctcatcacagccaccatcac
KDM5B	ctgcagccagaaggagatg	gggagacaccaacacagacc
MKI67	caagcactttggagagcaaa	gacacacacattgtcctcagc
CDC7	tgacaattaggggatccagag	ttgtgctggaacttctttgc
E2F8	gccaccaactatgactcacc	ctttgggtgtcacaggaacag
CDK1	ggaaggggttcctagtactgc	cctgcataagcacatcctga
UHRF1	atctgctgtcaggagctggt	aaaggatctgtccaggcagt
CCNA1	aaggagtgtgcgtcaggact	accctgtaaatgcagcaagg
CCNB1	catggtgcactttcctcctt	caggtgctgcataactggaa
CCND1	cgtggcctctaagatgaagg	ccacttgagcttgttcacca
CCNE1	cccacagagacagcttggat	tctttggtggagaaggatgg
E2F1	tgcagagcagatggttatgg	ctcagggcacaggaaaacat
RPLP0	ccttgtctgtggagacggattac	gccaagaaggccttgacctt

Supplementary Table S2. List of antibodies used in western blot analysis

Antibody	Clone number	Company
EHMT1	Ab135487	Abcam
EHMT2	C6H3	Cell signaling Technology
Histone H3	D1H2	Cell signaling Technology
Tri-Methyl-Histone H3 (Lys4)	C42D8	Cell signaling Technology
Di-Methyl-Histone H3 (Lys9)	D85B4	Cell signaling Technology
Tri-Methyl-Histone H3 (Lys27)	C36B11	Cell signaling Technology
EZH1	D7D5D	Cell signaling Technology
EZH2	D2C9	Cell signaling Technology
SET1A	D3V9S	Cell signaling Technology
MLL1	D2M7U	Cell signaling Technology
MLL2	D6X2E	Cell signaling Technology
WDR5	D9E11	Cell signaling Technology
WDR82	D2I3B	Cell signaling Technology
KDM5A (JARID1A)	D28B10	Cell signaling Technology
KDM4B (JMJD2B)	D7E6	Cell signaling Technology
KDM7C (PHF2)	D45A2	Cell signaling Technology
β-Actin	13E5	Cell signaling Technology

Supplementary Table S3. List of siRNAs used in the study

Gene Name	Company (siRNA IDs)	Sense (5' → 3')
MLL1	Bioneer (Custom siRNA)	AAG AAG UCA GAG UGC GAA GUC
MLL2	Bioneer (Custom siRNA)	GAA GGG CAU CGG GUG CUA UAU
WDR5	Bioneer (Custom siRNA)	GCU CAG AGG AUA ACC UUG U
WDR82	Bioneer (80335-1)	CUG AGU UGU UUA CAC UGA U

Supplementary Table S4. List of cell cycle genes whose H3K4me3 levels at the promoter regions were significantly reduced in response to metformin

Chr	Start	End	Distance to TSS[a]	Symbol	Read count[b] (Metformin)	Read count[b] (Control)	-log10 (p-value)
chr18	39535190	39538280	0	PIK3C3	343	572	17.61
chr7	86780543	86785001	0	DMTF1	1363	1719	15.57
chr12	66218207	66226958	0	HMGA2	2043	2458	15.44
chr12	111176663	111180680	103	PPP1CC	825	1091	13.57
chr2	234759577	234763179	33	HJURP	793	1012	10.57
chr10	94352901	94356194	76	KIF11	481	655	9.91
chr21	34915386	34918273	36	SON	756	954	9.40
chr7	35840986	35844294	390	SEPT7	442	600	9.05
chr3	172466851	172468191	-284	ECT2	169	282	9.04
chr15	40453253	40456270	43	BUB1B	701	887	9.02
chr10	62538251	62542968	0	CDK1	746	936	9.00
chr15	91535996	91537126	755	PRC1	182	293	8.61
chr5	159848839	159852531	0	PTTG1	784	969	8.44
chr6	160148699	160151762	167	WTAP	1007	1207	8.30
chr4	68409248	68411186	70	CENPC	224	337	7.98
chr12	6603217	6605069	0	NCAPD2	735	907	7.92
chr2	135676404	135680264	12	CCNT2	1014	1203	7.73
chr22	22218960	22221492	478	MAPK1	560	713	7.73
chr12	131357141	131359904	358	RAN	439	577	7.48
chr20	54963722	54967307	44	AURKA	754	920	7.46
chr2	172014611	172017008	402	TLK1	416	549	7.37
chr4	17812616	17816363	180	NCAPG	923	1097	7.24
chr7	36429423	36432102	0	ANLN	784	948	7.22
chr5	89705860	89707582	-257	CETN3	237	343	7.08
chr3	160117451	160122453	0	SMC4	1743	1948	6.77
chr2	111432164	111437312	0	BUB1	1087	1260	6.70
chr5	169010765	169014364	127	SPDL1	810	963	6.54
chr10	12235140	12240984	0	CDC123	939	1097	6.38
chr6	52149890	52151643	-211	MCM3	126	205	6.31
chr3	134200920	134204814	0	CEP63	888	1038	6.14
chr17	57228865	57232562	238	SKA2	1013	1169	6.12
chr7	40171889	40175930	0	MPLKIP	704	842	6.11
chr2	201390904	201393816	39	SGO2	592	722	6.09
chr9	6413462	6416427	311	UHRF2	670	795	5.48

chr12	12868197	12878157	0	CDKN1B	3346	3554	5.43
chr14	45719933	45724164	0	MIS18BP1	555	671	5.41
chr1	192778176	192779494	7	RGS2	166	242	5.27
chr14	105487785	105491325	-360	CDCA4	833	962	5.24
chr6	158244651	158247566	357	SNX9	655	774	5.22
chr15	52859175	52861165	48	ARPP19	461	566	5.15
chr5	36876972	36880035	111	NIPBL	837	961	5.04
chr10	124913906	124917106	146	BUB3	540	648	5.02
chr5	75011205	75013203	110	POC5	319	408	4.90
chr5	131892636	131895174	20	RAD50	518	622	4.85
chr1	32479905	32483771	610	KHDRBS1	829	947	4.76
chr2	99795287	99799966	0	MITD1	849	968	4.75
chr17	43021699	43026743	0	KIF18B	850	965	4.57
chr6	24773966	24778642	0	GMNN	1260	1387	4.50
chr3	49446435	49449021	407	RHOA	625	728	4.49
chr3	136468107	136470514	731	STAG1	379	464	4.33
chr11	108093556	108096120	0	ATM	573	667	4.21
chr16	72127772	72129987	0	TXNL4B	658	756	4.21
chr1	100596633	100600737	0	SASS6	742	844	4.19
chr10	95256493	95259159	104	CEP55	475	564	4.17
chr1	63987302	63990860	0	ITGB3BP	617	712	4.14
chr8	90993449	90997462	0	NBN	886	991	4.12
chr1	91965478	91969016	0	CDC7	589	681	4.06
chr14	50359821	50364501	85	ARF6	873	976	4.05
chrX	119691478	119694871	0	CUL4B	425	507	3.94
chr1	1405903	1407039	-125	ATAD3B	268	339	3.91
chr19	17378236	17379785	0	BABAM1	321	395	3.88
chr9	106854254	106859031	0	SMC2	736	830	3.87
chr6	33359586	33364063	273	KIFC1	1131	1236	3.85
chr2	97001872	97003529	388	NCAPH	248	314	3.75
chr17	57694595	57701692	0	CLTC	2024	2141	3.73
chr3	57261869	57263925	104	APPL1	306	376	3.72
chr20	31408107	31411850	408	MAPRE1	901	996	3.71
chr20	4667658	4671224	501	PRNP	294	362	3.65
chr12	49107177	49110513	268	CCNT1	1200	1298	3.56
chr18	77745574	77748363	169	TXNL4A	501	577	3.44
chr11	19260438	19262459	48	E2F8	217	276	3.42
chr14	61200796	61204154	0	MNAT1	573	650	3.39
chr13	73302194	73303278	152	BORA	167	221	3.36

chr15	41625098	41627324	206	NUSAP1	535	610	3.35
chr7	39990930	39995301	971	CDK13	786	869	3.34
chr13	33161381	33163257	817	PDS5B	242	302	3.34
chr15	50716199	50719112	0	USP8	646	722	3.24
chr15	59397337	59399298	53	CCNB2	387	454	3.23
chr14	99947772	99950743	33	CCNK	954	1036	3.17
chr7	92466045	92467333	-104	CDK6	279	339	3.16
chr13	114239745	114240437	0	TFDP1	74	113	3.15
chr11	18547279	18549756	0	TSG101	364	427	3.13
chr19	4910135	4913239	0	UHRF1	388	453	3.12
chr10	129921727	129924615	0	MKI67	560	630	3.10
chrX	154299776	154301760	81	BRCC3	246	301	3.05
chr20	30327091	30330503	187	TPX2	1610	1696	3.02
chr2	28974696	28978212	70	PPP1CB	567	636	3.02

[a] TSS stands for transcription start site

[b] Read count frequency of H3K4me3 in cells treated with and without metformin

Supplementary Table S5. H3K4me3 alteration at the promoter regions of CDKN1A (p21) and CDKN1B (p27) in response to metformin

Chr	Start	End	Distance ToTSS[a]	Symbol	Metformin[b]	Control[b]	-log10(p-value)
hr6	36649470	36650385	2983	CDKN1A	71	73	0.46
chr6	36648424	36648971	1937	CDKN1A	114	98	0.31
chr6	36648424	36648971	1937	CDKN1A	114	98	0.31
chr6	36646572	36648070	85	CDKN1A	584	391	5.00
chr6	36646572	36648070	85	CDKN1A	584	391	5.00
chr6	36646572	36648070	85	CDKN1A	584	391	5.00
chr6	36646572	36648070	85	CDKN1A	584	391	5.00
chr6	36646572	36648070	85	CDKN1A	584	391	5.00
chr6	36645574	36649016	0	CDKN1A	856	598	5.81
chr6	36645574	36649016	0	CDKN1A	856	598	5.81
chr6	36645574	36649016	0	CDKN1A	856	598	5.81
chr6	36645574	36649016	0	CDKN1A	856	598	5.81
chr6	36645574	36649016	0	CDKN1A	856	598	5.81
chr6	36645574	36649016	0	CDKN1A	856	598	5.81
chr6	36645574	36649016	0	CDKN1A	856	598	5.81
chr6	36645574	36649016	0	CDKN1A	856	598	5.81
chr6	36645574	36649016	0	CDKN1A	856	598	5.81
chr6	36645522	36646347	0	CDKN1A	80	64	0.38
chr6	36645522	36646347	0	CDKN1A	80	64	0.38
chr12	12867637	12868089	-2213	CDKN1B	77	57	0.51
chr12	12867457	12867884	-2418	CDKN1B	67	47	0.60

[a] TSS stands for transcription start site

[b] Read count frequency

Supplementary Table S6. List of cell cycle genes whose mRNA levels were downregulated by greater than or equal to 1.5 fold in response to metformin

Gene Symbol	Description	FC[a]
PSRC1	proline and serine rich coiled-coil 1	-2.59
ASPM	abnormal spindle microtubule assembly	-2.47
SGO2	shugoshin 2	-2.13
NUF2	NUF2, NDC80 kinetochore complex component	-2.08
NDC80	NDC80, kinetochore complex component	-2.03
DBF4	DBF4 zinc finger	-2.01
SMC4	structural maintenance of chromosomes 4	-2.00
RACGAP1	Rac GTPase activating protein 1	-2.00
KIF11	kinesin family member 11	-1.99
E2F8	E2F transcription factor 8	-1.92
DSCC1	DNA replication and sister chromatid cohesion 1	-1.90
CENPE	centromere protein E	-1.89
CDC27	cell division cycle 27	-1.89
CKAP2	cytoskeleton associated protein 2	-1.88
ITGB3BP	integrin subunit beta 3 binding protein	-1.87
NCAPG	non-SMC condensin I complex subunit G	-1.85
CCNE2	cyclin E2	-1.85
MKI67	marker of proliferation Ki-67	-1.83
CENPA	centromere protein A	-1.83
CENPX	centromere protein X	-1.83
PLK1	polo like kinase 1	-1.79
KIF18B	kinesin family member 18B	-1.79
BIRC5	baculoviral IAP repeat containing 5	-1.79
SPC25	SPC25, NDC80 kinetochore complex component	-1.78
MIS18BP1	MIS18 binding protein 1	-1.78
ESCO2	establishment of sister chromatid cohesion N-acetyltransferase 2	-1.78
KIF20B	kinesin family member 20B	-1.77
CCNA2	cyclin A2	-1.77
H2AFX	H2A histone family member X	-1.77
CDKN3	cyclin dependent kinase inhibitor 3	-1.76
KNL1	kinetochore scaffold 1	-1.76
TXNL4B	thioredoxin like 4B	-1.76
CCNF	cyclin F	-1.75
PPP1R1C	protein phosphatase 1 regulatory inhibitor subunit 1C	-1.75

DLGAP5	DLG associated protein 5	-1.74
MCM7	minichromosome maintenance complex component 7	-1.74
DCLRE1A	DNA cross-link repair 1A	-1.72
FAM83D	family with sequence similarity 83 member D	-1.72
NCAPD2	non-SMC condensin I complex subunit D2	-1.71
MCM4	minichromosome maintenance complex component 4	-1.70
SKA3	spindle and kinetochore associated complex subunit 3	-1.70
CDK1	cyclin dependent kinase 1	-1.69
FBXO5	F-box protein 5	-1.68
CDC6	cell division cycle 6	-1.68
E2F2	E2F transcription factor 2	-1.67
BORA	bora, aurora kinase A activator	-1.66
NEK2	NIMA related kinase 2	-1.66
CENPF	centromere protein F	-1.65
MAD2L1	mitotic arrest deficient 2 like 1	-1.65
LRRCC1	leucine rich repeat and coiled-coil centrosomal protein 1	-1.64
ECT2	epithelial cell transforming 2	-1.63
CDC7	cell division cycle 7	-1.63
HJURP	Holliday junction recognition protein	-1.63
MTBP	MDM2 binding protein	-1.62
AURKA	aurora kinase A	-1.61
SPAG5	sperm associated antigen 5	-1.61
HELLS	helicase, lymphoid-specific	-1.61
UHRF1	ubiquitin like with PHD and ring finger domains 1	-1.60
UBE2S	ubiquitin conjugating enzyme E2 S	-1.60
CCNB1	cyclin B1	-1.59
PTTG1	pituitary tumor-transforming 1	-1.59
E2F6	E2F transcription factor 6	-1.58
ZWINT	ZW10 interacting kinetochore protein	-1.58
SPDYA	speedy/RINGO cell cycle regulator family member A	-1.57
RBL1	RB transcriptional corepressor like 1	-1.57
ERCC6L	ERCC excision repair 6 like, spindle assembly checkpoint helicase	-1.57
NCAPH	non-SMC condensin I complex subunit H	-1.57
CDC45	cell division cycle 45	-1.56
MCM2	minichromosome maintenance complex component 2	-1.56
GMNN	geminin, DNA replication inhibitor	-1.56
CDCA8	cell division cycle associated 8	-1.55
CCPG1	cell cycle progression 1	-1.55

HMGA2	high mobility group AT-hook 2	-1.54
TFDP2	transcription factor Dp-2	-1.54
OIP5	Opa interacting protein 5	-1.53
CDC25A	cell division cycle 25A	-1.53
SYCP2	synaptonemal complex protein 2	-1.53
ANAPC10	anaphase promoting complex subunit 10	-1.53
DDIAS	DNA damage induced apoptosis suppressor	-1.52
SUV39H2	suppressor of variegation 3-9 homolog 2	-1.52
THAP5	THAP domain containing 5	-1.52
KNSTRN	kinetochore localized astrin/SPAG5 binding protein	-1.51
HAUS3	HAUS augmin like complex subunit 3	-1.51
SKA2	spindle and kinetochore associated complex subunit 2	-1.51
BUB1	BUB1 mitotic checkpoint serine/threonine kinase	-1.50

[a] FC indicates the ratio of mRNA levels in cells treated with metformin relative to those without metformin

Supplementary Table S7. List of cell cycle genes in which both H3K4me3 and mRNA were downregulated in response to metformin

Symbol	Description	FC[a]	*P*-value[b]
SGO2	shugoshin 2	-2.13	6.09
SMC4	structural maintenance of chromosomes 4	-2.00	6.77
KIF11	kinesin family member 11	-1.99	9.91
E2F8	E2F transcription factor 8	-1.92	3.42
ITGB3BP	integrin subunit beta 3 binding protein	-1.87	4.14
NCAPG	non-SMC condensin I complex subunit G	-1.85	7.24
MKI67	marker of proliferation Ki-67	-1.83	3.10
KIF18B	kinesin family member 18B	-1.79	4.57
MIS18BP1	MIS18 binding protein 1	-1.78	5.41
TXNL4B	thioredoxin like 4B	-1.76	4.21
NCAPD2	non-SMC condensin I complex subunit D2	-1.71	7.92
CDK1	cyclin dependent kinase 1	-1.69	9.00
BORA	bora, aurora kinase A activator	-1.66	3.36
ECT2	epithelial cell transforming 2	-1.63	9.04
CDC7	cell division cycle 7	-1.63	4.06
HJURP	Holliday junction recognition protein	-1.63	10.57
AURKA	aurora kinase A	-1.61	7.46
UHRF1	ubiquitin like with PHD and ring finger domains 1	-1.60	3.12
PTTG1	pituitary tumor-transforming 1	-1.59	8.44
NCAPH	non-SMC condensin I complex subunit H	-1.57	3.75
GMNN	geminin, DNA replication inhibitor	-1.56	4.50
HMGA2	high mobility group AT-hook 2	-1.54	15.44
SKA2	spindle and kinetochore associated complex subunit 2	-1.51	6.12
BUB1	BUB1 mitotic checkpoint serine/threonine kinase	-1.50	6.70

[a] FC indicates the ratio of mRNA levels in cells treated with metformin relative to those without.

[b] *P*-value indicates -log10(ratio of H3K4me3 read intensity in cells treated with metformin relative to those without metformin).

Supplementary Table S8. List of genes whose chromatin accessibility was significantly changed at promoter regions in response to metformin

Chr	Start[a]	End[a]	Length	Distance to TSS[b]	Gene Symbol	Log2Ratio
14	100375515	100375767	252	702	WARS	-2.15
2	105455571	105456035	464	-1112	LOC105373529	-1.97
9	114396940	114397202	262	1480	AKNA	-1.70
1	109214825	109215170	345	1104	SARS	-1.63
12	57516920	57517158	238	-3671	MBD6	-1.56
14	77181417	77181988	571	-57	TMEM63C	-1.49
5	74762568	74762953	385	-2669	NSA2	-1.45
4	74097642	74098030	388	1444	CXCL2	-1.41
8	24955922	24956875	953	471	NEFL	-1.36
4	90127129	90127669	540	5	CCSER1	-1.35
4	145481561	145482009	448	479	SMAD1	-1.35
17	55264704	55265128	424	-44	HLF	-1.33
20	380615	381184	569	270	TRIB3	-1.32
20	13221560	13222006	446	12	ISM1	-1.29
1	119712584	119712998	414	-976	LOC105378937	-1.25
22	41367181	41367603	422	59	TEF	-1.21
10	133159992	133160591	599	-176	KNDC1	-1.19
6	40587473	40588028	555	-286	LRFN2	-1.17
21	15064161	15064507	346	1569	NRIP1	-1.16
6	27814058	27814444	386	-793	HIST1H2BM	-1.16
20	63176432	63176823	391	-1873	MIR124-3	-1.15
8	119415973	119416618	645	-17	NOV	-1.12
10	87659625	87660446	821	-1090	LOC105378411	-1.12
19	18385985	18386379	394	24	GDF15	-1.10
5	75510805	75511139	334	1009	COL4A3BP	-1.10
13	110819467	110819862	395	-2785	LOC105370363	-1.10

7	150992718	150993104	386	1855	NOS3	-1.09
X	118495476	118496085	609	-129	DOCK11	-1.08
14	51093844	51094165	321	1700	TRIM9	-1.08
7	128783285	128783747	462	66	TRP-AGG2-8	-1.06
5	56816502	56817104	602	1730	MAP3K1	-1.06
X	129654369	129655086	717	229	APLN	-1.06
5	11903822	11904765	943	-250	CTNND2	-1.05
1	161539856	161540574	718	-26	TRN-GTT1-1	-1.05
3	75672383	75672768	385	294	LINC00960	-1.05
12	111597293	111597684	391	2188	ATXN2	-1.03
10	68827036	68827733	697	-153	STOX1	-1.02
5	6766529	6766930	401	819	LOC102724943	-1.02
2	131363728	131364221	493	184	WTH3DI	-1.01
9	69324547	69325366	819	384	FAM189A2	-1.01
9	12814160	12814845	685	-113	LURAP1L-AS1	-1.00
11	414900	415292	392	2358	SIGIRR	1.00
3	149702809	149703438	629	150	WWTR1	1.03
16	30051518	30051886	368	1310	FAM57B	1.08
19	3176983	3177556	573	-1469	S1PR4	1.10
12	43792707	43793286	579	-2768	TMEM117	1.11
15	89823364	89823767	403	2116	LOC105370965	1.11
9	93670624	93671045	421	2416	LOC101929519	1.16
1	16980659	16981093	434	767	MFAP2	1.19
16	90031517	90031927	410	-1821	GAS8-AS1	1.23
1	51970452	51970847	395	-2761	RNA5SP48	1.24
1	23556705	23557117	412	439	LOC105376859	1.33
14	50038390	50038998	608	1190	LOC100506499	1.46
1	8009143	8010756	1613	-2920	LOC105376694	1.75

[a] Based on the Genome Reference Consortium (GRC) 38

[b] TSS stands for transcription start site

Supplementary Table S9. Cox proportional hazards analysis of survival according to MLL2 expression in lung adenocarcinoma (N = 27)

Histology	MLL2	HR	95% CI	P-value
Overall	Normal	1.00		
Survival[a]	Overexpressed	1.08	0.92 - 1.27	0.35
Recurrence-free	Normal	1.00		
Survival[b]	Overexpressed	1.32	1.08 - 4.72	0.02

[a] Adjusted for age, sex, recurrence, and pathologic stage

[b] Adjusted for sex and pathologic stage

Abbreviations: Adeno, adenocarcinoma; squamous, squamous cell carcinoma; HR, hazard ratio; CI, confidence interval
